# Global genomic diversity of *Pseudomonas aeruginosa* in bronchiectasis

**DOI:** 10.1016/j.jinf.2024.106275

**Published:** 2024-09-16

**Authors:** N.E. Harrington, A. Kottara, K. Cagney, M.J. Shepherd, E.M. Grimsey, T. Fu, R.C. Hull, C.E. Chong, K.S. Baker, D.Z. Childs, J.L. Fothergill, J.D. Chalmers, M.A. Brockhurst, S. Paterson

**Affiliations:** a Institute of Infection, Veterinary and Ecological Sciences, University of Liverpool, L69 3BX, UK; b Division of Evolution and Genomic Sciences, School of Biological Sciences, University of Manchester, M13 9NT, UK; c Division of Molecular and Clinical Medicine, University of Dundee, Ninewells Hospital and Medical School, Dundee, DD1 9SY, UK; d Department of Genetics, University of Cambridge, CB2 3EH, Cambridge, UK; e Department of Animal and Plant Sciences, University of Sheffield, S10 2TN, Sheffield, UK

**Keywords:** *Pseudomonas aeruginosa*, bronchiectasis, whole genome sequencing, genetic variation, drug resistance, bacterial, bacterial infections, lung

## Abstract

**Background:**

*Pseudomonas aeruginosa* is the most common pathogen in the bronchiectasis lung, associated with worsened outcomes. *P. aeruginosa* genomic studies in this context have been limited to single-country, European studies. We aimed to determine strain diversity, adaptation mechanisms, and AMR features to better inform treatment.

**Methods:**

*P. aeruginosa* from 180 bronchiectasis patients in 15 countries, obtained prior to a phase 3, randomised clinical trial (ORBIT-3), were analysed by whole-genome sequencing. Phylogenetic groups and sequence types were determined, and between versus within patient genetic diversity compared using Analysis of Molecular Variance (AMOVA). The frequency of AMR-associated genes and mutations was also determined.

**Results:**

2,854 *P. aeruginosa* isolates were analysed, predominantly belonging to phylogenetic group 1 (83%, n = 2,359). Genetic diversity was far greater between than within patients, responsible for >99.9% of total diversity (AMOVA: phylogroup 1: df = 145, *P* < 0.01). Numerous pathways were under selection, some shared with CF (e.g., motility, iron acquisition), some unique to bronchiectasis (e.g., novel efflux pump PA1874). Multidrug resistance features were also frequent.

**Conclusions:**

We present a 10-fold increase in the availability of genomic data for *P. aeruginosa* in bronchiectasis, highlighting key distinctions with cystic fibrosis and potential targets for future treatments.

## Introduction

Non-cystic fibrosis bronchiectasis (hereafter referred to as bronchiectasis) is a chronic respiratory disease of unknown cause that occurs in a broad demographic and is not monogenic^1^. It is defined by abnormal, permanent dilation of the bronchi that results in impaired clearance of mucus from the airways, leading to chronic microbial infections and inflammation^4^. Patients experience ongoing symptoms of a cough, sputum production, and frequent respiratory infections that drive progressive lung function decline and reduced quality of life^2,3^

The dominant bacterial pathogen is *Pseudomonas aeruginosa*, associated with an ~7-fold increased risk of hospitalisation and 3-fold increased risk of mortality^4^. A recent analysis of the European bronchiectasis registry, involving 16,963 patients from 28 countries, identified *P. aeruginosa* infection in ~25% of patients^5^. There was marked regional variation among these infections, with more than 50% of cases in Southern Europe and lower rates in Northern Europe. *P. aeruginosa* has also been shown to be the dominant pathogen in the United States^6^, China^7^, India and Australia^8^.

Until recently, bronchiectasis has been a neglected disease with limited research. There are few genomic studies characterising the associated *P. aeruginosa* infections, particularly in comparison to cystic fibrosis (CF), a rare genetic cause of bronchiectasis. To date, there have been only two single-country genomic epidemiology studies solely focused on *P. aeruginosa* in bronchiectasis, both in Northern Europe. A study in Germany investigated 130 *P. aeruginosa* genomes from 110 adult patients attending a single bronchiectasis clinic, and found infections were caused by diverse sequence types (STs) representative of global diversity^9^. The incidence of multiple patients with an infection caused by the same ST was rare. Similarly, a UK study of 189 *P. aeruginosa* genomes from 91 adult patients attending 16 clinics showed a high diversity of STs^10^. However, this study also reported that ~30% of patients had mixed ST infections, suggesting multiple acquisition events in these patients.

In the more extensively studied context of CF, *P. aeruginosa* is known to undergo a characteristic suite of genomic changes during infection that enable adaptation to the infection environment ^11^. This drives *P. aeruginosa* genetic diversification through mutations affecting antimicrobial resistance (AMR), biofilm formation, and regulatory systems involved in a range of functions including virulence, which have been implicated in prolonging infection and reducing the effectiveness of antimicrobial treatments, directly impacting patient health^11,12^. The lack of genomic data for *P. aeruginosa* infections in bronchiectasis means it is unclear whether such evolutionary diversification occurs^10^. Taking into consideration the more variable aetiology, disease phenotypes, and treatment responses^13^, it is likely that the evolutionary pathways of *P. aeruginosa* in bronchiectasis may be less predictable than in CF.

*P. aeruginosa* genomic studies of a larger number of bronchiectasis patients from different global regions, and increased sampling of clones per infection, are urgently required to guide improved treatment of this neglected disease. To address this, we used genome sequencing to analyse a unique global collection of 2,854 *P. aeruginosa* bronchiectasis isolates obtained from 180 patients prior to the start of the ORBIT-3 Phase 3 clinical trial^14^. Our findings advance understanding of strain diversity, the likelihood of multiple acquisition events, the prevalence of antimicrobial resistance determinants, and the evolutionary mechanisms underpinning chronic infection in bronchiectasis.

## Methods

### Patient samples

Sputum samples were collected prior to the start of the ORBIT-3 phase 3, randomised clinical trial for inhaled liposomal ciprofloxacin in bronchiectasis^14^. To be eligible, patients were required to have a history of chronic *P. aeruginosa* infection and have experienced a minimum of two pulmonary exacerbations requiring antibiotic treatment in the last year^14^. Samples from a cohort of 180 patients, attending clinics in five global regions (Fig S1; USA & Canada, Western Europe, Central & Eastern Europe, Australia & New Zealand, Other), were used for the present study, across both the treatment and placebo groups (119 and 61 patients respectively). Patient histories were not available (including previous antibiotic treatments and duration of infection).

### *Pseudomonas aeruginosa* isolation and growth

Sputum samples were stored at -80 °C for *P. aeruginosa* isolation to be performed as part of this study. An equal volume of Sputasol solution (SR0233, Oxoid) was added to each sample, mixed until liquefaction was complete, and then incubated in a 37 °C shaking incubator (240 rpm) for 30 min. Subsequently, 100 μl was spread onto Cetrimide Agar (22470, NutriSelect® Plus) plates and incubated in a static incubator at 37 °C for 24 - 48 h. These populations were further streaked onto Cetrimide Agar plates and incubated in a static incubator at 37°C for 24 – 48 h, and 96 *P. aeruginosa* colonies were then isolated from each population. These selected colonies were grown in King’s B medium consisting of 20 g l^-1^ Bacto proteose peptone No.3 (Gibco), 1.5 g l^-1^ Potassium phosphate dibasic trihydrate (P5504, Sigma), 1.5 g l^-1^ Magnesium sulfate heptahydrate (M1880, Sigma) and 10 g l^-1^ Glycerol (49770, Honeywell) in a static incubator at 37 °C for 48 h. The presence of *P. aeruginosa* was confirmed using polymerase chain reaction (PCR) and primers targeting the species-specific 16S rRNA gene of *P. aeruginosa*. The primer sequences used were: forward, 5’ - GGG GGA TCT TCG GAC CTC A - 3’; reverse, 5’ - TCC TTA GAG TGC CCA CCC G - 3’. The PCR protocol included the following thermocycling program: initial denaturation at 95 °C for 5 min, followed by 35 cycles of denaturation at 95 °C for 20 s, annealing at 58 °C for 20 s, extension at 72 °C for 40 s, and a final extension at 72 °C for 10 min.

### DNA extraction and sequencing

16 *P. aeruginosa* isolates per patient were selected for sequencing. DNA extraction was performed following the Quick-DNA Fecal/Soil Microbe Kit (Zymo Research) on a KingFisher Flex instrument (Thermo Fisher Scientific). The eluted DNA was quantified using a Qubit Flex Fluorometer (Thermo Fisher Scientific) and then standardised to 7.7 ng μl^-1^ using a MANTIS Liquid Handler (Formulatrix). Library preparation was performed using 20 ng DNA following the NEBNext Ultra II FS DNA Library Prep Kit for Illumina (New England Biosciences) on a Mosquito HV liquid handling instrument (SPT Labtech), miniaturized to 0.1 recommended volume. Library fragments were amplified and 10 bp index sequences (Integrated DNA Technologies) were incorporated using PCR.

Libraries were quantified using a Qubit Flex Fluorometer and size analysis was performed on a Fragment Analyser (Agilent). The average library length and concentration of each library was used to pool libraries in an equimolar manner using a Mosquito X1 (SPTLabtech). The final pooled libraries were cleaned and concentrated using AMPure XP beads (Beckman Coulter). The average length of the final pool of libraries was analysed using a Bioanalyzer (Agilent) and the concentration quantified using a Qubit. Samples were then sequenced on an Illumina NovaSeq 6000 150 bp paired-end run by the Centre for Genomics Research, University of Liverpool, who trimmed the raw fastq files using Cutadapt v1.2.1^15^.

### Bioinformatic analysis

Full analysis pipeline and package information are detailed in the supplementary information. Briefly, all reads were quality checked, *de novo* assembly performed (median number of contigs = 80; see Table S1) and genomes annotated. Genome assemblies and annotations were used to construct the pangenome and build core single nucleotide polymorphism (SNP) phylogenies, perform multi locus sequence type (MLST) analysis, and screen for structural variants, prophages, plasmid replicons, CRISPR-Cas subtypes, all defence systems, genomic islands, core SNPs, and AMR genes and mutations.

### Reference strains and gene information

Four reference strain genomes were included in this work: *P. aeruginosa* PAO1 (NCBI, GCF_000006765.1), *P. aeruginosa* PA14 (NCBI, GCF_000014625.1), *P. aeruginosa* LESB58 (NCBI, GCF_000026645.1) and *P. aeruginosa* PA7 (NCBI, GCF_000017205.1). All gene information was sourced from pseudomonas.com^16^.

### Statistical analysis

All statistical analyses were performed using R v4.3.1. The *pegas* package^17^ was used to conduct AMOVAs (1000 permutations) and to calculate nucleotide diversity (π).

## Results

**Figure 1:**
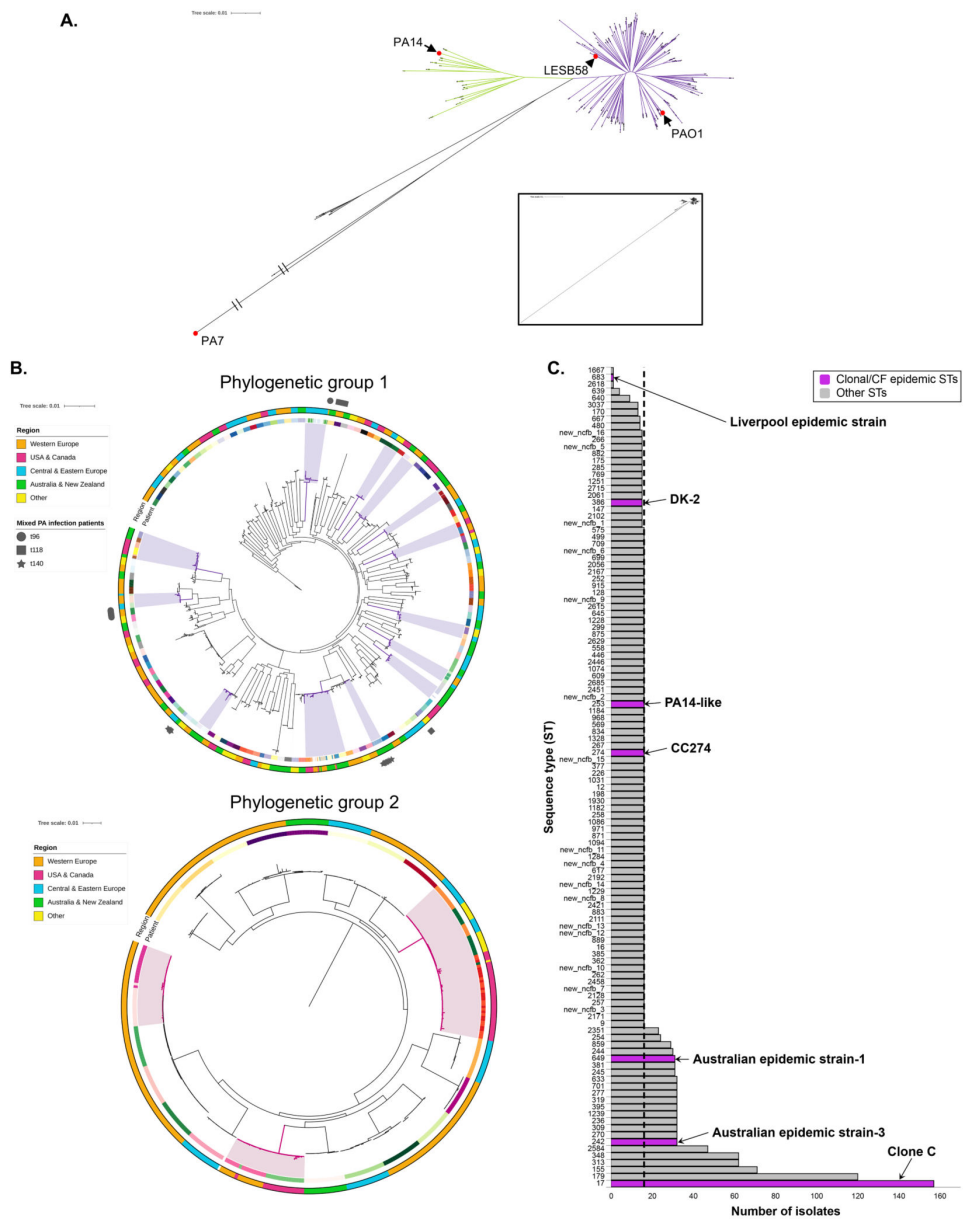
**(A)** Unrooted core single nucleotide polymorphism (SNP)-based phylogenetic tree of *Pseudomonas aeruginosa* isolates from people with non-cystic fibrosis (CF) bronchiectasis and 4 common reference strains: PAO1, LESB58, PA14 and PA7 (circled and labelled). Each group is denoted by coloured branches (group 1: right, purple, group 2: left, green, group 3+: down, black). **(B)** Core SNP phylogenetic trees of the two dominant phylogenetic groups, group 1 (PAO1-like) and group 2 (PA14-like). The inner coloured rings show the patient each isolate was taken from, and the outer coloured rings show the global region each patient was located (see legend). The shapes outside the group 1 tree show the mixed *P. aeruginosa* infection isolates (see legend). The highlighted regions show clusters of patients whose isolates branched together. **(C)** *P. aeruginosa* sequence types (STs) identified from multi-locus sequence type (MLST) analysis of isolates from people with bronchiectasis. The isolate frequency of all confirmed STs, both known and new (as labelled), is shown. The dashed line indicates 16 isolates, which was the total sequenced from each patient, indicating STs present in a single patient. Clonal STs and CF epidemic STs are show in purple and labelled.

We sequenced ~16 *P. aeruginosa* isolates from each of 180 patients attending clinics in five global regions, made up of 15 countries including Australia, Taiwan, the USA, Latvia, and the UK (Fig S1). Patients ranged in age from 21 to 87 years old (median = 68), and 67% were female (Fig S1). A core SNP phylogeny of all isolates was constructed (Fig 1A; based on 4,344 core genes). The majority of isolates either belonged to phylogenetic group 1 (83%) or phylogenetic group 2 (14%; Table S2). We observed strong clustering of isolates by patient but no clear evidence of clustering by global region (Fig 1B), suggesting genomic diversity is not predominantly driven by geography. Clonal STs, such as the common strain clone C, and known CF epidemic STs were infrequent, found in 7% (n = 12) and 3% (n = 6) of patients respectively (Fig 1C; Table S4). However, the 5 most frequent STs found were among the twenty most common *P. aeruginosa* STs globally ^18^. Incidences where multiple patients were found to carry the same ST were from different clinics, often in different global regions (Fig 1B; Table S3), suggesting that transmissible strains do not play a major role in bronchiectasis. There was also limited evidence for coexistence of different *P. aeruginosa* strains within single patients indicating mutliple infection events are rare; only 2% (n = 3) were found to have mixed ST infections (Fig S2).

**Figure 2:**
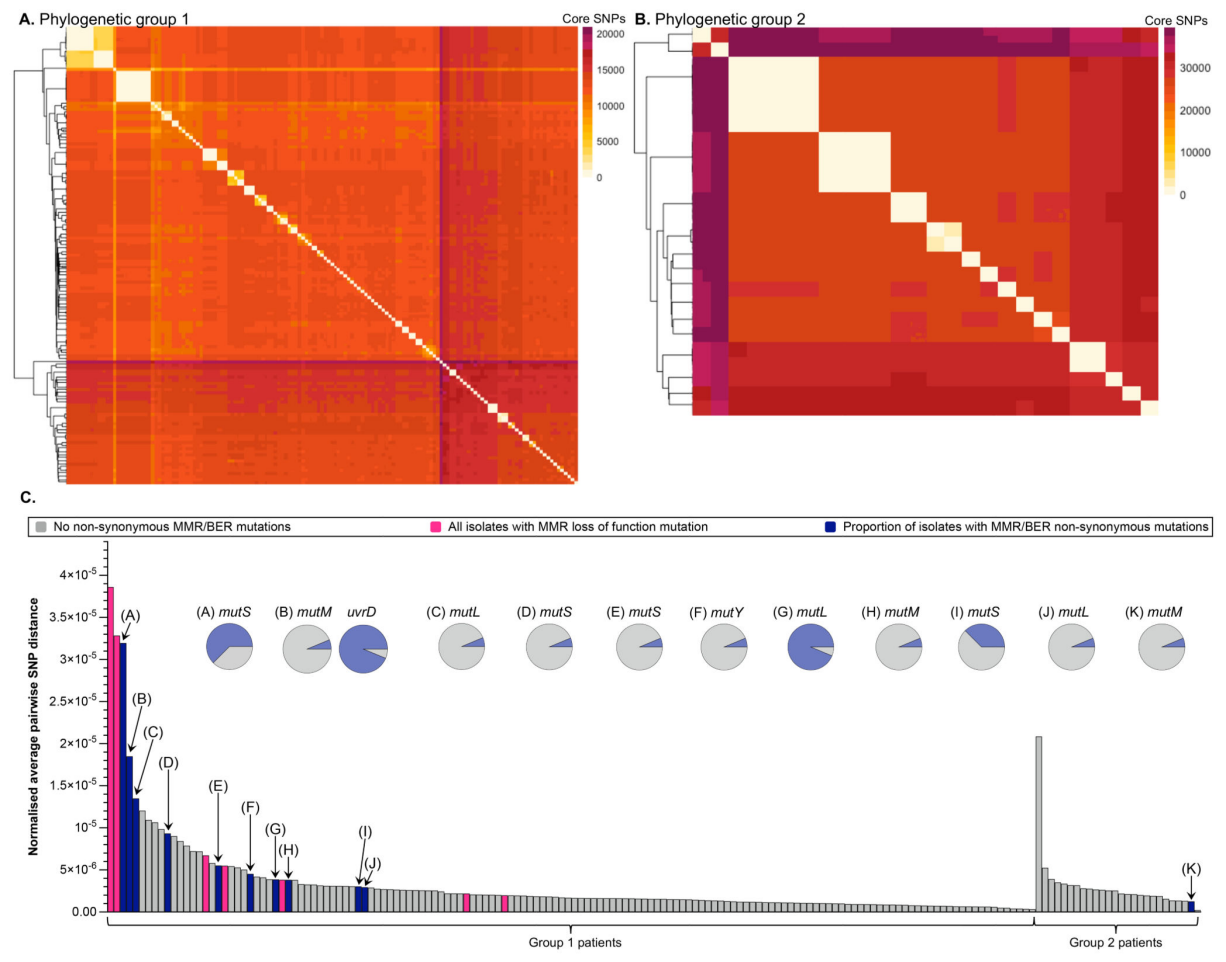
**(A,B)** Heatmaps showing the core single nucleotide polymorphism (SNP) pairwise distance matrix of phylogenetic group 1 (A) and phylogenetic group 2 (B) *Pseudomonas aeruginosa* bronchiectasis isolates. Higher nucleotide diversity was found in group 2 isolates than group 1 (group 2 π=6.11×10^-3^; average 1 SNP per 164 bases; group 1 π=2.93x10 ^-3^; average 1 SNP per 342 bases). The fill represents the number of core SNPs between each isolate (see legend), and the light coloured diagonal line shows same patient isolate comparisons. The dendrograms show isolate clustering based on core SNP pairwise distances. **(C)** The normalised average pairwise SNP distance for each patient, calculated as the average pairwise core SNP distance between isolates from the same patient divided by the number of core nucleotides. Groups shown are phylogenetic groups. The patients with all isolates carrying a loss of function mutation in DNA mismatch repair (MMR) genes are highlighted in pink. No loss of function mutations in base excision repair (BER) genes were detected. Patients with non-synonymous mutations between isolates (i.e. polymorphic) are highlighted in blue and labelled with letters corresponding to pie charts that show the proportion of sequenced isolates carrying the mutation (blue = with mutation, grey = without).

*P. aeruginosa* genetic diversity was far greater between than within patients, accounting for >99.9% of total diversity in both group 1 and group 2 isolates (AMOVA: group 1: df = 145, *P* < 0.01, group 2: df = 25, *P* < 0.01). The average pairwise core SNP distance between co-existing isolates of single ST infections (Fig 2A) did not significantly differ between groups (T test: *t*_45.04_ = 0.26, *P* = 0.80). There were 117 group 1 patients (80%) and 23 group 2 patients (88%) with an average pairwise core SNP distance <20 between isolates (Fig 2B), further supporting between patient differences as the major source of genetic diversity in bronchiectasis infections. Nonetheless, there was evidence of within-patient diversification in remaining patients, often associated with mutations in genes encoding DNA mismatch repair (MMR) or break excision repair (BER), likely causing hypermutation (Fig 2C), as well as structural variants detected among isolates from 34 patients (Table S5).

Variation in gene content also contributed to *P. aeruginosa* genomic diversity, and similarly showed significantly greater variation between than within patients (1-sample t-tests: group 1 *t*_145_= -725.53, *P* < 0.01; group 2 *t*_25_= -202.15, *P* < 0.01; Fig S5). There was extensive gene content variation in both phylogroups (group 1: 4,580 core genes and 14,138 accessory genes, n=2,349 isolates; group 2: 4,627 core genes and 6,817 accessory genes, n=415 isolates), and accessory genome sizes within patients ranged from 1,701 genes to 59 genes (Fig S5). Mobile genetic elements played a role in generating this within-patient diversity. Prophage regions (20 to 433 genes in size; mean = 135 genes) were observed in all isolates (1 to 9 regions per genome; mean = 3; Table S6), and coexisting isolates from the same patient often varied in prophage number (50%; n = 89 patients) and/or the gene content of prophage regions (86%; n = 153 patients). Additionally, functional CRISPR-Cas subtypes were found in 57% of patients (n = 103 patients; Table S6), predominantly the I-F subtype (44% isolates; n = 1257 isolates; Fig S3), which was the most common defence system across all isolates (Fig S4), and a number of genomic islands (Table S6; Table S7). Plasmids were comparatively much rarer, detected in only 53 isolates from 6 patients, but variable plasmid carriage between coexisting isolates was associated with the highest level of within patient gene content variation observed (Fig S5).

**Figure 3:**
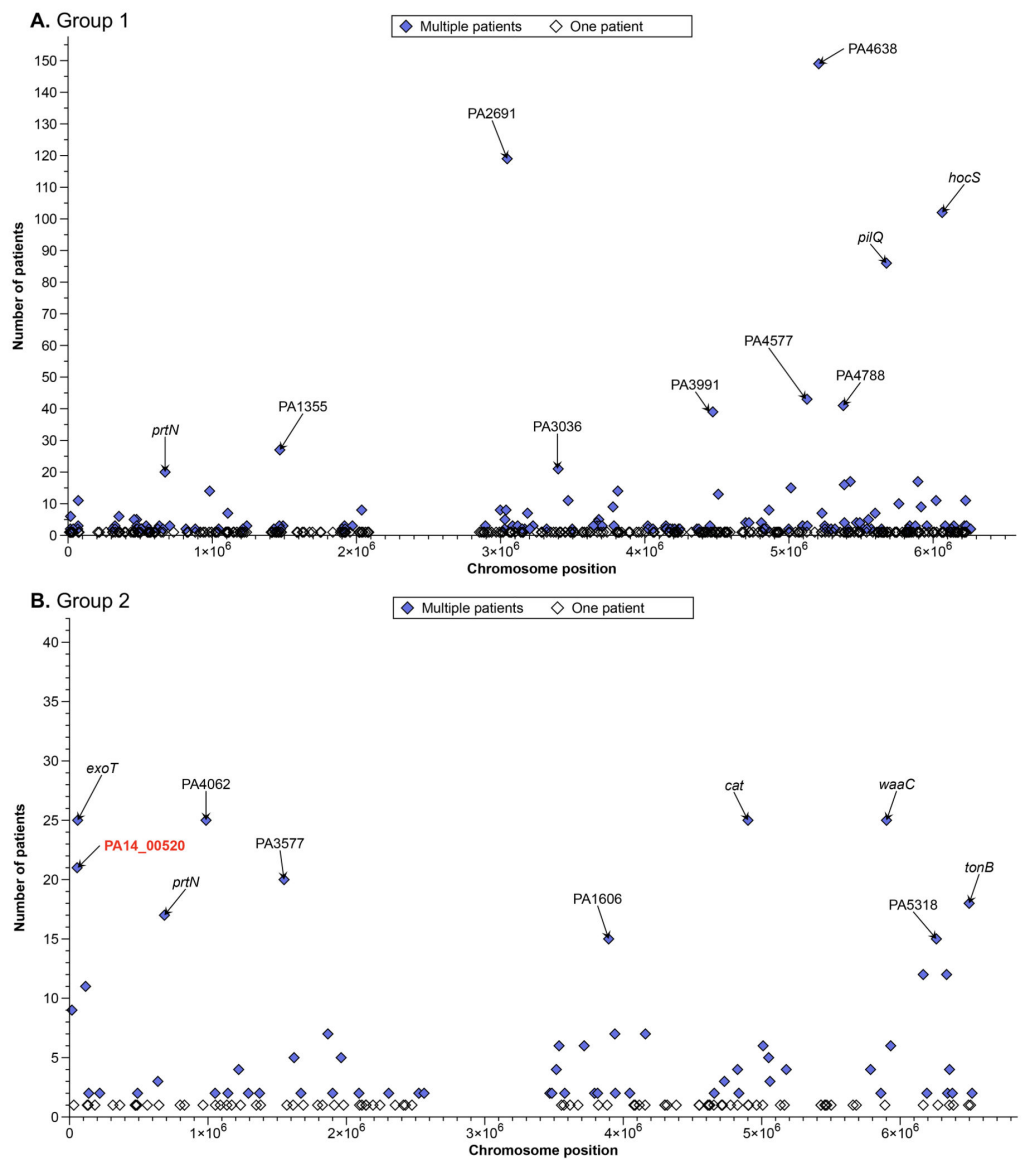
The number of bronchiectasis patients with at least one *Pseudomonas aeruginosa* isolate with a high impact single nucleotide polymorphism (SNP) (likely causing loss of function) in a gene. Each gene is represented by a data point. Phylogenetic group 1 **(A)** and group 2 **(B)** are shown on separate graphs as different reference strains were used for each (group 1: PAO1, group 2: PA14). The mutations in multiple patients are shown in blue, and the 10 most frequent amongst patients are labelled. The PA14 locus tags have been converted to PAO1 where possible, the red highlighted gene is not present in PAO1. A large chromosomal region with no core SNPs in either group is shown, indicative of large deletions occurring in a proportion of isolates; similar deletions have previously been associated with increased AMR in *P. aeruginosa* clinical isolates^22^.

We next investigated genes that had accumulated loss of function (LoF) mutations in multiple patients, indicating functions potentially involved in bronchiectasis lung adaptation. LoF mutations causing interruption of start/stop codons were found in 135 genes in group 1 (Fig 3A) and 60 genes in group 2 (Fig 3B). Among these were phylogenetically independent SNPs that had become fixed in multiple patients (i.e., parallel evolution; Table S8). The majority of genes gained LoF mutations in <10 patients. However, 21 genes and 13 genes among group 1 and group 2 patients, respectively, gained these mutations more frequently (Fig 3). Of these, ~50% were hypothetical (11 genes in group 1 and 6 genes in group 2) and three were common to both phylogroups. This included the positive regulator of pyocin expression^19^ *prtN* (37 patients; 21%), cell-surface receptor gene *tonB*, linked to iron uptake, biofilm formation and quorum sensing^20^ (29 patients; 17%), and the fosfomycin transporter-encoding gene *glpT* (29 patients; 17%), mutations in which have previously been shown to confer resistance to fosfomycin^21^. LoF in genes associated with motility (*pilQ*), metabolism (*hocS, cntI, aruD*), and heme-aquisition (*hasD*) were also common in group 1 (Fig 3A). In group 2, 96% of patients (25; Fig 3B) were found to carry LoF mutations in the lipopolysaccharide biosynthesis gene *waaC*, chloramphenicol acetyltransferase *cat*, and virulence-associated gene *exoT*. LoF mutations in these three genes were not observed in group 1, indicating potentially distinct routes of adaptation between the main phylogroups.

**Figure 4:**
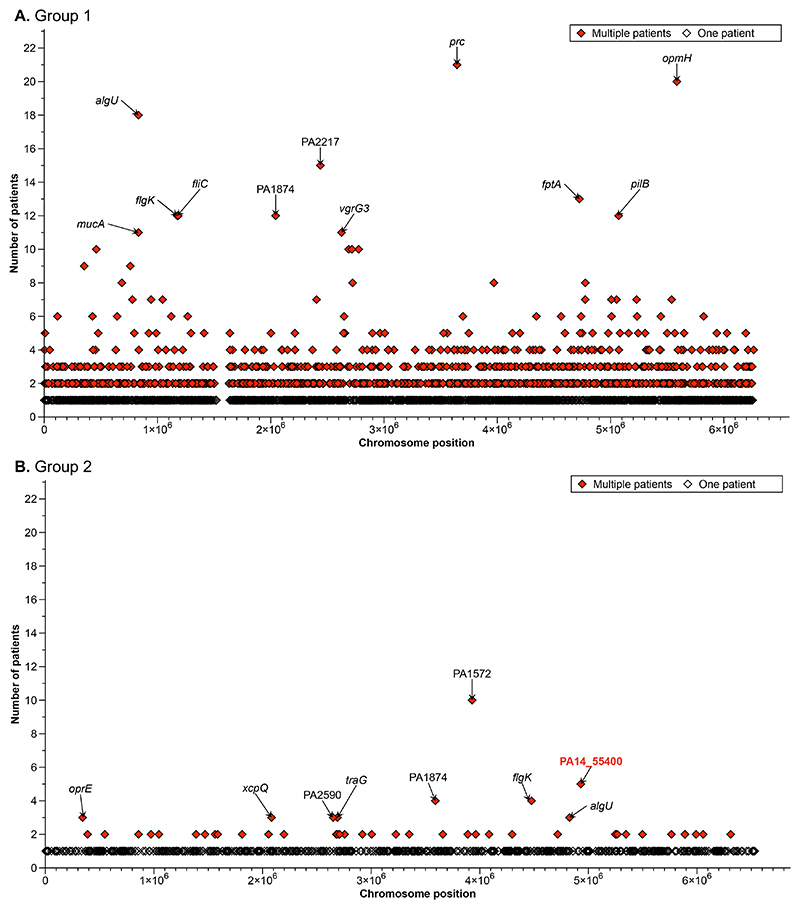
Within patient polymorphism. Each data point represents a gene with non-synonymous mutations between *Pseudomonas aeruginosa* isolates from the same bronchiectasis patient. They are filled if a mutation in the gene separates isolates in more than one patient (see legend). The y axis shows the number of patients with mutations between their sequenced isolates in each gene. The dataset has been split into phylogenetic group 1 **(A)** and group 2 **(B)** as different reference strains were used for each (group 1: PAO1, group 2: PA14). The top genes are labelled. The PA14 locus tags have been converted to PAO1 where possible for group 2, the red highlighted gene is not present in PAO1.

To determine putative targets of diversifying selection within patients, we then identified genes with non-synonymous polymorphisms across multiple patients. Three polymorphic genes were frequent in group 1 and group 2 (Fig 4; Table S8): a novel efflux pump gene (PA1874; 9% of patients), sigma factor-encoding gene *algU* (PA0762; 12% of patients) and the flagellar-associated gene *flgK* (PA1086; 9% of patients). Additionally, in group 1, polymorphic genes associated with a range of key functions were identified including motility (*fliC, chpA* and *pilB*), cell envelope (*oprE, opmH, oprD, pagL, migA*), alginate production (*mucA, algG*), and iron acquisition (*fptA, pvdL, pvdS, pchE, hemA, pvdP*), as well as *pdtA*, encoding a filamentous haemagluttinin linked with adhesion and virulence. In group 2, the majority of genes were found to be polymorphic in ≤ 2 patients (Fig 4B). However, one gene, PA1572, also known as *nadK1*, which encodes an ATP-NAD kinase associated with response to reactive oxygen species^23^, was found to be polymorphic in 38% of patients (Fig 4B).

**Figure 5:**
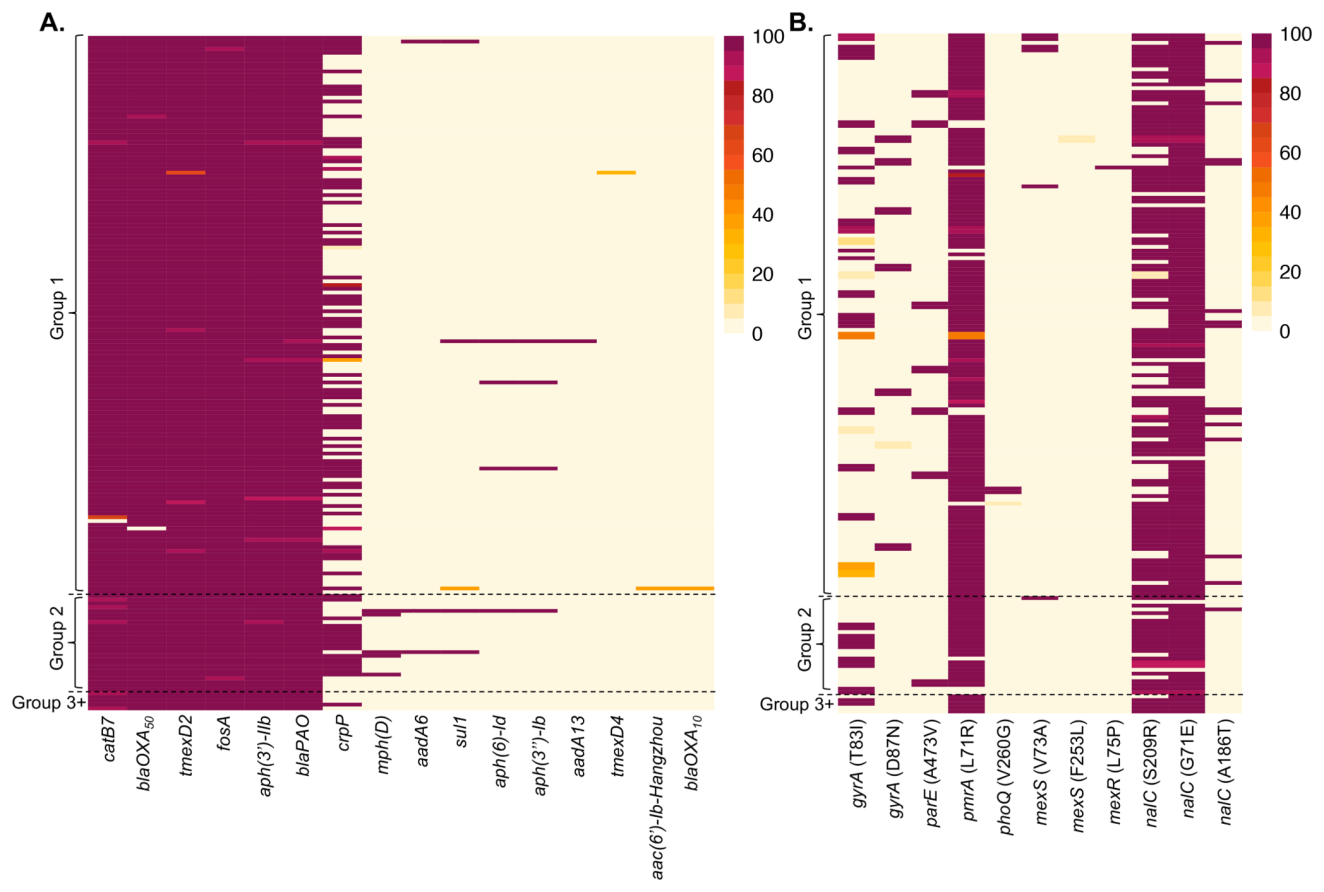
**(A)** The presence/absence of antimicrobial resistance (AMR) genes in each bronchiectasis *Pseudomonas aeruginosa* isolate was identified using ResFinder. The patient without any OXA-50 beta-lactamases detected was found to have a large deletion in all isolates in this region (Fig S6). **(B)** The presence of AMR-associated mutations in each isolate based on Comprehensive Antibiotic Resistance Database (CARD) predictions, identified using RGI. In both heatmaps, each row represents a patient, and the fill colour shows the percentage of isolates from each patient with the gene or mutation (see keys). Groups shown are the phylogenetic groups.

The abundance and distribution of *P. aeruginosa* AMR determinants between and within infections in bronchiectasis is poorly understood. We used the ResFinder and CARD databases to identify 16 AMR genes and 11 AMR-associated mutations in our collection (Fig 5). The incidence of within patient polymorphism for AMR was rare, with most genes and mutations either present or absent in all isolates per patient. Six of the AMR genes were detected in almost all isolates, highlighting the high likelihood of multidrug resistance, including two beta-lactamase genes typically seen in the *P. aeruginosa* core genome (bla_OXA-50_ and bla_PAO_), two variants of the multidrug transporter *mexD*, an aminoglycoside phosphotransferase (*aph(3’)-IIb*), and the chloramphenicol acetyltransferase gene *catB7*. Additionally, the enzyme-encoding gene *fosA* conferring fosfomycin resistance was found in all patients (Fig 5A).

Among the AMR genes variable in their presence, likely representing gene gain events, the most common was presence of the ciprofloxacin modifying enzyme *crpP* (49% of patients; Fig 5A). The remaining variable genes were detected in ≤ 5 patients (Fig 5A), indicating horizontal gene acquisition is unlikely to be the main driver of AMR. Multiple variable genes were typically found in single patients, for example 6 isolates from a single patient had an OXA-10-like beta-lactamase and an aminoglycoside acetyltransferase gene (*aac(6’)-Ib-Hangzhou*) typically seen in *Acinetobacter baumanii* (Fig 5A)^24^. The presence of AMR-associated SNPs was also variable between patients (Fig 5B), most commonly arising in regulators of multidrug efflux systems (*nalC, mexS, mexR*) associated with upregulation, the response regulator *pmrA* (L71R; >80% of patients) associated with colistin resistance, and mutations associated with ciprofloxacin resistance (in *gyrA* and *parE*). AMR mutations in *gyrA* were mutually exclusive and were the most variable between patients, and among coexisting isolates (T83I in 124 patients, and D87N in 14 patients; Fig 5B).

## Discussion

*P. aeruginosa* is the most common cause of respiratory infections in bronchiectasis worldwide, contributing to higher morbidity and mortality rates. However, *P. aeruginosa* genomic diversity in bronchiectasis is poorly understood. Our study provides a 10-fold increase in the availability of genomic data, expanding patient sampling beyond existing studies in Europe^9,10^. We have shown infections are predominantly caused by distinct STs, with low incidence of CF epidemic clones and mixed ST infections, and little evidence for geographic impact. Consistently, *P. aeruginosa* genetic diversity was greater between patients than within patients. We identified *P. aeruginosa* genes and functions undergoing parallel evolution in multiple patients that are likely to be associated with adaptation to the bronchiectasis lung, many overlapping with those commonly seen in CF and other contexts such as chronic obstructive pulmonary disease (COPD), as well as some that appeared to be bronchiectasis-specific.

*P. aeruginosa* epidemic strains are a major factor in the epidemiology of CF infections. However, CF epidemic strains appear to be rare in bronchiectasis. Combined with the high diversity of STs across patients, consistent with the prior single-country studies^9,10^, our findings indicate that highly-transmissible strains play only a minor role in bronchiectasis infection worldwide. Additionally, in contrast to the smaller UK study (189 isolates; 91 patients) which found 29% of patients had multilineage *P. aeruginosa* infections^10^, we did not observe high incidence of mixed *P. aeruginosa* infections. This implies that cohorting and isolation procedures to prevent dissemination of and superinfection by transmissible clones, more common in CF and Western countries, may not be required for bronchiectasis *P. aeruginosa* infection control.

Our findings provide strong evidence that *P. aeruginosa* undergoes adaptation to the bronchiectasis lung environment. Our analysis revealed a suite of pathways under selection in multiple patients. Some functions gaining LoF mutations were common to both bronchiectasis and CF, including motility (*flgK, fliC* and *pilQ*) and iron acquisition (*tonB* and *fptA*). In contrast, other mutational targets appeared to be more common in bronchiectasis than in CF, including mutations affecting pyocin production and resistance genes, indicating bacteriocin-mediated interference competition may be less intense compared to CF, and a novel efflux pump gene, PA1874. There is limited research on the function of PA1874, although mutations have been linked to increased tobramycin resistance during biofilm infection^25^. Our findings of distinct evolutionary paths in bronchiectasis suggest that, despite shared features with CF, there exist potentially important differences in the genomic adaptation that may be of clinical importance.

Although adaptation to the bronchiectasis lung was not found to be associated with extensive *P. aeruginosa* genetic diversity within patients, we observed some common causes of within patient diversification. The majority were similar to adaptations seen in CF ^11^, including polymorphism in genes involved in motility and alginate production. The mucoidy regulator AlgU was found to be a common target of diversifying selection across both phylogenetic groups. A recent study showed that *algU* was more likely be mutated in CF isolates than non-CF^18^. It was also reported that *mucA* was a key target of pathoadaptive mutations in *P*. aeruginosa^18^, which we found to be frequently polymorphic among group 1 isolates. The instances of higher SNP distances within patients were often linked with hypermutator-associated mutations, suggesting that also in common with CF, the emergence of hypermutation can drive rapid within patient diversification. Differences in gene content driven by variable mobile genetic element carriage, including prophages and less commonly plasmids, also led to substantial genetic diversity between isolates within some patients.

*P. aeruginosa* infections in bronchiectasis are often treated intensively with antibiotics after initial colonisation^26^, followed by long-term suppressive antibiotic therapy and targeted antibiotic treatment for exacerbations, and as such patients experience a range of antibiotic classes during their lifecourse. This has been shown to impact the resistome and to drive increase multidrug resistance^27^. We observed frequent *P. aeruginosa* regulatory mutations likely to cause upregulation of multidrug efflux systems. This included mutations affecting MexAB-OprM that have not been highlighted previously. More drug-specific resistance mechanisms were also found, including fluoroquinolone resistance genes (*crpP*) and mutations in the targeted topoisomerases (*gyrA* and *parE*), and fosfomycin resistance genes (*fosA*) and mutations (in *glpT*). Whilst homologues of *fosA* were found to be present in 98.8% of *P. aeruginosa* published genomes as of 2017^28^, implicating it as an intrinsic resistance mechanism, mutations in *glpT* have been shown to confer much higher levels of fosfomycin resistance^29^. The parallel evolution of *glpT* LoF in our cohort suggests fosfomycin is unlikely to be an effective treatment option for bronchiectasis. Overall we show that AMR features are typically present or absent in all isolates per patient and multidrug resistance features are common, suggesting that combined antibiotic therapies may often be necessary in bronchiectasis.

Taking into consideration the serious impact of *P. aeruginosa* infection upon patient health, improving treatments for bronchiectasis lung infections is a high priority. This study provides an unprecedented global genomic resource improving our knowledge and understanding of *P. aeruginosa* genetic diversity and adaptation in bronchiectasis. Our findings highlight important differences between bronchiectasis and CF infections, notably the relatively minor role that transmissible strains play in bronchiectasis, and highlight potential targets and considerations for the development of treatment regimens.

## Supplementary Material

Supplementary information

Table S1

Table S5

Table S7

## Data Availability

All sequencing data (reads and assemblies) are available at the European Nucleotide Archive (ENA) (accession number: PRJEB65845).
