# Supplementary material for: Global genomic diversity of Pseudomonas aeruginosa in bronchiectasis
Source: J Infect. Author manuscript; Available in PMC 2026 Feb 28. (PMC7618805; doi:10.1016/j.jinf.2024.106275)
Supplement: Supplementary information [file EMS212666-supplement-Supplementary_information.docx]

**Supplementary Methods**

**Genome assembly, annotation and MLST analysis**

All reads were then quality checked with FastQC v0.11.9^1^, and *de novo* assembly performed using Unicycler v0.5.0^2^. Each genome was annotated with Bakta v1.6.0^3^, and quality checked using the quality control (QC) pre-processing script as part of Panaroo v1.2.10^4^, QUAST v5.2.0^5^ and Busco v5.4.3^6^, and any isolates with poor assemblies were removed. Panaroo QC was also used to detect samples contaminated with reads from other species (indicative of initial sample contamination) or non-*P. aeruginosa* isolates, which were then removed from the analyses. All remaining assemblies were sequence typed with mlst v2.11^7^, using the PubMLST database (<https://pubmlst.org/>)^8^.

**Pangenome and phylogeny construction**

The pangenome of each sample group of interest: all isolates, each phylogroup and individual patients, was constructed using Panaroo v1.2.10^9^ with mafft alignment. The core genome produced was then used to determine the core SNP phylogeny for each group using SNP-sites v2.5.1^10^. Maximum likelihood phylogenetic trees were then constructed based on these using IQ-Tree v2.0.0.3^11^ (1000 bootstraps) with ModelFinder for model selection^12^ and ascertainment bias correction.

**Structural variants**

Short-read structural variant detection among each set of isolates within a patient was conducted with GRIDSS2^13^, using the most continguous genome from these isolates as a reference with the remaining isolates mapped against this reference as described below. Contigs less than 10 Kbp and regions less than 5 Kbp from the end of a reference contig were excluded to reduce false positives. Potential breakpoints were filtered for those in which there were at least 50% of reads associated with the variant in one sample and that were also polymorphic among isolates within a patient.

**Prophage and plasmid detection**

To screen for viral DNA and detect prophage regions in each genome, VirSorter v1.0.5^4^ was used. Viral sequences detected in each assembled genome assigned to prophage category 1 (#4) and prophage category 2 (#5) were considered prophage regions, selected at random for confirmation using PHASTEST^15-17^. To detect possible plasmids in assemblies, Abricate v0.7^8^ was used to identify any plasmid replicons against the PlasmidFinder database^19^.

**Defence system detection**

To identify defence systems present in each isolate, DefenseFinder ^20-22^ was used to screen all genomes. To further investigate the presence of intact, functional CRISPR-Cas subtypes in assembled genomes, CRISPRCasTyper ^23^.

**Genomic islands**

Genomic islands were detected in genome annotations against the phylogenetically closest reference strain supported (as discussed in the main text; PAO1 for group 1, PA14 for group 2), using IslandViewer4^24^.

**Mutation diversity**

To investigate mutational diversity, all SNPs in each genome were first identified against the annotation file for the closest reference strain for each phylogenetic group (group 1: *P. aeruginosa* PAO1, group 2: PA14, group 3+: PA7) using Snippy v4.6.0^25^. The ‘snippy-core’ script, part of Snippy, was then used to determine core SNPs amongst the different groups of isolates described. This identified all SNPs in core sites amongst each group of isolates (e.g. phylogenetic groups/patient populations). SNP pairwise distance matrices were then produced using snp-dists v0.8.2 and visualised as heatmaps using the *pheatmap* package in R v4.3.1^26^. SnpEff v5.0e^27^ was subsequently used to annotate the core SNPs identified. Comparisons to determine polymorphic genes and fixed mutations were performed using these outputs in R. Ancestral state reconstruction, under an equal-rates model, of loss of function core SNPs observed in multiple isolates was performed and mapped to the phylogeny using SIMMAP^28^ and the *phytools* package^29^ in R v4.3.1.

**Antimicrobial resistance genome features**

To detect the presence of antimicrobial resistance genes in each genome assembly, ResFinder 4.0^30^ was used. There was one isolate from a patient where OXA-50, expected in all *P. aeruginosa* genomes, was not detected by ResFinder, however visual inspection using IGV v2.16.1^31^ confirmed this was a false negative. To detect AMR-associated mutations, RGI was used against the Comprehensive Antibiotic Resistance Database (CARD)^32^.

**References**

1. Andrews, S. FastQC: a quality control tool for high throughput sequence data. https://www.bioinformatics.babraham.ac.uk/projects/fastqc/ (2018).

2. Wick, R. R., Judd, L. M., Gorrie, C. L. & Holt, K. E. Unicycler: Resolving bacterial genome assemblies from short and long sequencing reads. *PLoS Comput Biol* **13**, (2017).

3. Schwengers, O. *et al.* Bakta: rapid and standardized annotation of bacterial genomes via alignment-free sequence identification. *Microb Genom* **7**, 685 (2021).

4. Tonkin-Hill, G. *et al.* Producing polished prokaryotic pangenomes with the Panaroo pipeline. *Genome Biol* **21**, 1–21 (2020).

5. Mikheenko, A., Prjibelski, A., Saveliev, V., Antipov, D. & Gurevich, A. Versatile genome assembly evaluation with QUAST-LG. *Bioinformatics* **34**, i142–i150 (2018).

6. Simão, F. A., Waterhouse, R. M., Ioannidis, P., Kriventseva, E. V. & Zdobnov, E. M. BUSCO: assessing genome assembly and annotation completeness with single-copy orthologs. *Bioinformatics* **31**, 3210–3212 (2015).

7. Seemann, T. mlst GitHub. https://github.com/tseemann/mlst.

8. Jolley, K. A. & Maiden, M. C. J. BIGSdb: Scalable analysis of bacterial genome variation at the population level. *BMC Bioinformatics* **11**, (2010).

9. Tonkin-Hill, G. *et al.* Producing polished prokaryotic pangenomes with the Panaroo pipeline. *Genome Biol* **21**, 1–21 (2020).

10. Page, A. J. *et al.* SNP-sites: rapid efficient extraction of SNPs from multi-FASTA alignments. *Microb Genom* **2**, e000056 (2016).

11. Quang, B. *et al.* IQ-TREE 2: New Models and Efficient Methods for Phylogenetic Inference in the Genomic Era. doi:10.1093/molbev/msaa015.

12. Kalyaanamoorthy, S., Minh, B. Q., Wong, T. K. F., Von Haeseler, A. & Jermiin, L. S. ModelFinder: Fast Model Selection for Accurate Phylogenetic Estimates. *Nat Methods* **14**, 587 (2017).

13. Cameron, D. L. *et al*. GRIDSS2: comprehensive characterisation of somatic structural variation using single breakend variants and structural variant phasing. *Genome Biol* **22**, 1 (2012).

14. Roux, S., Enault, F., Hurwitz, B. L. & Sullivan, M. B. VirSorter: Mining viral signal from microbial genomic data. *PeerJ* **2015**, e985 (2015).

15. Zhou, Y., Liang, Y., Lynch, K. H., Dennis, J. J. & Wishart, D. S. PHAST: A Fast Phage Search Tool. *Nucleic Acids Res* **39**, W347 (2011).

16. Arndt, D. *et al.* PHASTER: a better, faster version of the PHAST phage search tool. *Nucleic Acids Res* **44**, W16 (2016).

17. Wishart, D. S. *et al.* PHASTEST: faster than PHASTER, better than PHAST. *Nucleic Acids Res* **51**, W443–W450 (2023).

18. Seemann, T. Abricate GitHub. https://github.com/tseemann/abricate.

19. Carattoli, A. *et al.* In silico detection and typing of plasmids using PlasmidFinder and plasmid multilocus sequence typing. *Antimicrob Agents Chemother* **58**, 3895–903 (2014).

20. Tesson, F. *et al*. Systematic and quantitative view of the antiviral arsenal of prokaryotes. *Nature Communications* **13**, 2561 (2022).

21. Abby, S., Néron, B., Ménager, H., Touchon, M. & Rocha, E. P. C. MacSyFinder: A Program to Mine Genomes for Molecular Systems with an Application to CRISPR-Cas Systems. *PloS one* **9**, 10 (2014).

22. Couvin, D. *et al*. CRISPRCasFinder, an update of CRISRFinder, includes a portable version, enhanced performance and integrates search for Cas proteins. *Nucleic Acids Res* **46**, W246-251 (2018).

23. Russel, J., Pinilla-Redondo, R., Mayo-Munoz, D., Shah, S. A. & Sorensen, S. J. CRISPRCasTyper: Automated Identification, Annotation, and Classification of CRISPR-Cas Loci**.** *The CRISPR Journal* **3**, 6 (2020).

24. Bertelli, C. *et al*. IslandViewer 4: Expanded prediction of genomic islands for larger-scale datasets. *Nucleic Acids Research* **45**, W1 (2017).

25. Seemann, T. Snippy: fast bacterial variant calling from NGS reads. https://github.com/tseemann/snippy (2015).

26. R Core Team. R: A Language and Environment for Statistical Computing. https://www.R-project.org/ (2023).

27. Cingolani, P. *et al.* A program for annotating and predicting the effects of single nucleotide polymorphisms, SnpEff:  SNPs in the genome of Drosophila melanogaster strain w1118; iso-2; iso-3. *Fly (Austin)* **6**, 80 (2012).

28. Bollback, J. P. SIMMAP: Stochastic character mapping of discrete traits on phylogenies. *BMC Bioinformatics* **7**, 1–7 (2006).

29. Revell, L. phytools: an R package for phylogenetic comparative biology (and other things). *Methods Ecol Evol* **3**, 217–223 (2011).

30. Bortolaia, V. *et al.* ResFinder 4.0 for predictions of phenotypes from genotypes. *Journal of Antimicrobial Chemotherapy* **75**, 3491 (2020).

31. Robinson, J. T. *et al.* Integrative Genomics Viewer. doi:10.1038/nbt.1754.

32. Alcock, B. P. *et al.* CARD 2023: expanded curation, support for machine learning, and resistome prediction at the Comprehensive Antibiotic Resistance Database. *Nucleic Acids Res* **51**, D690–D699 (2023).

**Supplementary Figures and Tables**

**Figure S1.** The demographic of bronchiectasis patients that sequenced *Pseudomonas aeruginosa* isolates were obtained from as part of the ORBIT3 clinical trial. **(A)** The distribution of ages at the start of the trial for all patients with available data. **(B)** The number of patients of each sex from available data. **(C)** The global regions represented by our sample set. The pie chart shows the proportion of patients attending clinics in each region, with the specific countries involved listed. The map shows these countries.

**Table S2.** The proportion of *Pseudomonas aeruginosa* isolates sequenced from people with bronchiectasis belonging to each phylogenetic group. Those in groups other than 1 and 2 have been grouped together, referred to as group 3+.

| **Phylogenetic group** | **Number of isolates** | **Patients (% (n))** |
| --- | --- | --- |
| 1 | 2359 | 82.78% (149) |
| 2 | 415 | 14.44% (26) |
| 3+ | 80 | 2.78% (5) |

**Figure S2.** Bronchiectasis patients found to have more than one *Pseudomonas aeruginosa* sequence type (ST), identified from multi-locus sequence type (MLST) analysis, represented in the 16 isolates sequenced**.** This is indicative of mixed-strain infection, and the proportion of isolates per ST identified in each patient is shown.

**Table S3.** The frequency of the 5 most prevalent sequence types (> 2 patients) amongst sequenced *Pseudomonas aeruginosa* isolates from people with bronchiectasis, and the global region they were sampled from. All are among the most common clones in the global *P. aeruginosa* population.

| **Sequence type (ST)** | **Number of isolates** | **Number of patients** | **Percentage of patients (n = 180)** | **Region** |
| --- | --- | --- | --- | --- |
| **179** | 120 | 8 | 4.44% | Western Europe, Australia & New Zealand, USA & Canada, Other |
| **155** | 71 | 5 | 2.78% | Western Europe, Australia & New Zealand, Other |
| **313** | 62 | 4 | 2.22% | USA & Canada, Central & Eastern Europe, Other |
| **348** | 62 | 4 | 2.22% | Australia & New Zealand, USA & Canada |
| **2584** | 47 | 3 | 1.67% | Central & Eastern Europe |

**Table S4.** The frequency of cystic fibrosis (CF)-associated transmissible strains, including CF epidemic sequence types and clone C, amongst sequenced *Pseudomonas aeruginosa* isolates from people with bronchiectasis, and the global region they were sampled from.

|  | **Sequence type (ST)** | **Number of isolates** | **Number of patients** | **Percentage of patients (n = 180)** | **Region** |
| --- | --- | --- | --- | --- | --- |
| **Clone C** | 17 | 157 | 10 | 5.56% | Western Europe, Australia & New Zealand, USA & Canada |
| **Liverpool epidemic strain (LES)** | 683 | 1 | 1 | 0.56% | Western Europe |
| **PA14-like** | 253 | 16 | 1 | 0.56% | Central & Eastern Europe |
| **Australian epidemic strain-1 (AES-1)** | 649 | 31 | 2 | 1.11% | Central & Eastern Europe, Other |
| **Australian epidemic strain-3 (AES-3)** | 242 | 32 | 2 | 1.11% | Australia & New Zealand |
| **DK-2** | 386 | 15 | 1 | 0.56% | Western Europe |
| **CC274** | 274 | 16 | 1 | 0.56% | Western Europe |

**Table S6.** The phylogenetic group (referred to as group) the isolates from each patient belong to, the number of genomic island regions detected in a representative isolate per patient (using IslandViewer; see Table S7 for full details), and the proportion of intact CRISPR-Cas subtypes found (using CRISPRCasTyper) and prophage regions detected (using VirSorter) amongst sequenced *Pseudomonas aeruginosa* isolates for each bronchiectasis patient. Each row represents a patient, with the patient code assigned in the first column. The fourth column shows the functional CRISPR-Cas subtype/s identified in isolates from that patient, and the percentage of isolates that carry it. The remaining columns show the percentage of isolates sequenced with each number of prophage regions (see column headings). Empty boxes are where there are no isolates from the patient with that number of prophage regions. The percentages are colour coded from highest percentage (dark green) to lowest (light green).

|  |  |  |  | **Number of prophage regions** | | | | | | | | |
| --- | --- | --- | --- | --- | --- | --- | --- | --- | --- | --- | --- | --- |
| **Patient** | **Group** | **Genomic islands** | **CRISPR-Cas subtype**  **(% isolates)** | **1** | **2** | **3** | **4** | **5** | **6** | **7** | **8** | **9** |
| t49 | 1 | 56 | I-F (100) | **100** |  |  |  |  |  |  |  |  |
| t133 | 1 | 30 | I-F (100) | **100** |  |  |  |  |  |  |  |  |
| p79 | 1 | 34 | I-F (100) | **100** |  |  |  |  |  |  |  |  |
| p14 | 1 | 48 | I-F (100) | **100** |  |  |  |  |  |  |  |  |
| t136 | 1 | 23 | I-F (100) | **100** |  |  |  |  |  |  |  |  |
| p43 | 1 | 21 | I-E (100) | **100** |  |  |  |  |  |  |  |  |
| t147 | 1 | 16 | I-F (100) | **100** |  |  |  |  |  |  |  |  |
| p74 | 1 | 28 | I-F (100) | **100** |  |  |  |  |  |  |  |  |
| t86 | 1 | 38 | I-F (100) | **100** |  |  |  |  |  |  |  |  |
| t82 | 1 | 28 | I-F (100) | **100** |  |  |  |  |  |  |  |  |
| p46 | 1 | 19 | I-F (100) | **100** |  |  |  |  |  |  |  |  |
| t44 | 1 | 34 | I-F (100) | **100** |  |  |  |  |  |  |  |  |
| t23 | 1 | 29 | I-F (100) | **100** |  |  |  |  |  |  |  |  |
| p26 | 2 | 16 | *None* | **100** |  |  |  |  |  |  |  |  |
| t90 | 2 | 30 | *None* | **100** |  |  |  |  |  |  |  |  |
| t79 | 2 | 20 | *None* | **100** |  |  |  |  |  |  |  |  |
| t58 | 2 | 50 | *None* | **100** |  |  |  |  |  |  |  |  |
| t81 | 1 | 56 | *None* |  | **100** |  |  |  |  |  |  |  |
| t92 | 1 | 29 | I-F (100) |  | **100** |  |  |  |  |  |  |  |
| p75 | 1 | 15 | *None* |  | **100** |  |  |  |  |  |  |  |
| t129 | 1 | 16 | I-F (96), *None* (4) |  | **100** |  |  |  |  |  |  |  |
| t13 | 1 | 70 | *None* |  | **100** |  |  |  |  |  |  |  |
| t158 | 1 | 32 | I-F (100) |  | **100** |  |  |  |  |  |  |  |
| t77 | 1 | 28 | *None* |  | **100** |  |  |  |  |  |  |  |
| t161 | 1 | 37 | I-C (100) |  | **100** |  |  |  |  |  |  |  |
| p28 | 1 | 62 | *None* |  | **100** |  |  |  |  |  |  |  |
| t154 | 1 | 23 | I-F (100) |  | **100** |  |  |  |  |  |  |  |
| p81 | 1 | 28 | *None* |  | **100** |  |  |  |  |  |  |  |
| t150 | 1 | 23 | *None* |  | **100** |  |  |  |  |  |  |  |
| t46 | 1 | 59 | I-F (100) |  | **100** |  |  |  |  |  |  |  |
| p38 | 1 | 36 | I-F (100) |  | **100** |  |  |  |  |  |  |  |
| p80 | 1 | 28 | I-F (100) |  | **100** |  |  |  |  |  |  |  |
| t51 | 1 | 44 | *None* |  | **100** |  |  |  |  |  |  |  |
| p77 | 1 | 9 | I-F (100) |  | **100** |  |  |  |  |  |  |  |
| p72 | 1 | 75 | I-F (100), I-E (100) |  | **100** |  |  |  |  |  |  |  |
| t172 | 1 | 31 | I-E (100) |  | **100** |  |  |  |  |  |  |  |
| p7 | 1 | 56 | I-F (100) |  | **100** |  |  |  |  |  |  |  |
| t149 | 1 | 16 | I-F (100) |  | **100** |  |  |  |  |  |  |  |
| t80 | 1 | 34 | I-E (100) |  | **100** |  |  |  |  |  |  |  |
| t127 | 1 | 69 | *None* |  | **100** |  |  |  |  |  |  |  |
| p33 | 1 | 26 | *None* |  | **100** |  |  |  |  |  |  |  |
| p76 | 1 | 32 | I-F (100) |  | **100** |  |  |  |  |  |  |  |
| t70 | 1 | 22 | I-F (100), I-E (100) |  | **100** |  |  |  |  |  |  |  |
| t5 | 1 | 20 | I-F (100) |  | **100** |  |  |  |  |  |  |  |
| p12 | 2 | 43 | I-C (100) |  | **100** |  |  |  |  |  |  |  |
| t72 | 2 | 32 | I-C (100) |  | **100** |  |  |  |  |  |  |  |
| t66 | 1 | 31 | *None* |  |  | **100** |  |  |  |  |  |  |
| t59 | 1 | 38 | I-E (100) |  |  | **100** |  |  |  |  |  |  |
| p10 | 1 | 51 | *None* |  |  | **100** |  |  |  |  |  |  |
| t130 | 1 | 26 | I-F (100) |  |  | **100** |  |  |  |  |  |  |
| t142 | 1 | 63 | *None* |  |  | **100** |  |  |  |  |  |  |
| t84 | 1 | 28 | *None* |  |  | **100** |  |  |  |  |  |  |
| t139 | 1 | 24 | I-F (100), I-C (100) |  |  | **100** |  |  |  |  |  |  |
| p61 | 1 | 19 | I-F (100) |  |  | **100** |  |  |  |  |  |  |
| p60 | 1 | 57 | *None* |  |  | **100** |  |  |  |  |  |  |
| t9 | 1 | 26 | *None* |  |  | **100** |  |  |  |  |  |  |
| t153 | 1 | 32 | I-E (100) |  |  | **100** |  |  |  |  |  |  |
| t125 | 1 | 35 | *None* |  |  | **100** |  |  |  |  |  |  |
| t33 | 1 | 11 | I-F (100) |  |  | **100** |  |  |  |  |  |  |
| p4 | 1 | 64 | I-F (100), I-C (100) |  |  | **100** |  |  |  |  |  |  |
| t54 | 1 | 33 | I-C (100) |  |  | **100** |  |  |  |  |  |  |
| t120 | 1 | 30 | I-F (100) |  |  | **100** |  |  |  |  |  |  |
| t68 | 1 | 26 | *None* |  |  | **100** |  |  |  |  |  |  |
| t99 | 1 | 57 | *None* |  |  | **100** |  |  |  |  |  |  |
| p57 | 1 | 22 | *None* |  |  | **100** |  |  |  |  |  |  |
| t6 | 1 | 36 | I-F (100), I-E (100) |  |  | **100** |  |  |  |  |  |  |
| t7 | 1 | 34 | I-F (100) |  |  | **100** |  |  |  |  |  |  |
| t10 | 2 | 46 | *None* |  |  | **100** |  |  |  |  |  |  |
| t89 | 2 | 49 | *None* |  |  | **100** |  |  |  |  |  |  |
| t155 | 1 | 83 | I-F (100), I-E (100) |  |  |  | **100** |  |  |  |  |  |
| t71 | 1 | 60 | *None* |  |  |  | **100** |  |  |  |  |  |
| t141 | 1 | 72 | *None* |  |  |  | **100** |  |  |  |  |  |
| t168 | 1 | 36 | *None* |  |  |  | **100** |  |  |  |  |  |
| p69 | 1 | 28 | I-F (100) |  |  |  | **100** |  |  |  |  |  |
| p42 | 1 | 93 | *None* |  |  |  | **100** |  |  |  |  |  |
| p23 | 1 | 38 | *None* |  |  |  | **100** |  |  |  |  |  |
| t152 | 1 | 49 | *None* |  |  |  | **100** |  |  |  |  |  |
| p82 | 1 | 47 | *None* |  |  |  | **100** |  |  |  |  |  |
| t109 | 1 | 57 | *None* |  |  |  | **100** |  |  |  |  |  |
| t78 | 2 | 50 | *None* |  |  |  | **100** |  |  |  |  |  |
| t94 | 1 | 63 | I-F (100) |  |  |  |  | **100** |  |  |  |  |
| t113 | 1 | 64 | I-F (100) |  |  |  |  | **100** |  |  |  |  |
| t156 | 1 | 34 | *None* |  |  |  |  | **100** |  |  |  |  |
| t122 | 1 | 67 | I-E (100) |  |  |  |  |  | **100** |  |  |  |
| p59 | 1 | 68 | *None* |  |  |  |  |  | **100** |  |  |  |
| t61 | 2 | 55 | *None* |  |  |  |  |  | **100** |  |  |  |
| p50 | 2 | 66 | I-E (100) |  |  |  |  |  | **100** |  |  |  |
| t85 | 1 | 38 | *None* |  |  |  |  |  |  | **100** |  |  |
| t87 | 1 | 39 | *None* |  |  | **6** | **94** |  |  |  |  |  |
| p21 | 1 | 31 | I-F (100) |  |  | **6** | **94** |  |  |  |  |  |
| p78 | 1 | 64 | *None* |  |  | **6** | **94** |  |  |  |  |  |
| t110 | 1 | 48 | I-F (94), I-E (100) |  |  | **6** |  | **94** |  |  |  |  |
| p70 | 1 | 60 | *None* |  |  |  | **6** | **94** |  |  |  |  |
| p40 | 1 | 23 | *None* |  | **6** | **94** |  |  |  |  |  |  |
| t143 | 1 | 30 | I-F (100) |  | **6** | **94** |  |  |  |  |  |  |
| t165 | 1 | 52 | *None* |  |  | **6** |  | **94** |  |  |  |  |
| t107 | 1 | 30 | I-F (100) | **94** |  | **6** |  |  |  |  |  |  |
| p67 | 1 | 29 | I-F (6) |  |  | **94** | **6** |  |  |  |  |  |
| t1 | 1 | 56 | I-F (100) |  | **6** |  | **94** |  |  |  |  |  |
| t128 | 1 | 26 | I-F (100) |  |  | **6** | **94** |  |  |  |  |  |
| t132 | 1 | 51 | I-F (100) |  |  |  | **6** | **94** |  |  |  |  |
| t157 | 1 | 33 | I-F (100) | **6** | **94** |  |  |  |  |  |  |  |
| t151 | 1 | 38 | I-F (100) |  |  | **6** | **94** |  |  |  |  |  |
| p63 | 2 | 19 | I-F (100) |  | **94** | **6** |  |  |  |  |  |  |
| t55 | 2 | 39 | *None* |  | **6** | **94** |  |  |  |  |  |  |
| t12 | 2 | 50 | *None* |  | **6** | **94** |  |  |  |  |  |  |
| p49 | 2 | 46 | I-C (100) |  | **6** | **94** |  |  |  |  |  |  |
| t174 | 2 | 54 | *None* |  |  |  |  |  | **6** | **94** |  |  |
| t144 | 3+ | 33 | I-E (100) |  | **6** | **94** |  |  |  |  |  |  |
| t116 | 3+ | 22 | *None* |  |  |  | **6** | **94** |  |  |  |  |
| t123 | 1 | 29 | I-F (100) |  | **7** | **93** |  |  |  |  |  |  |
| t101 | 1 | 72 | *None* |  |  |  | **8** | **92** |  |  |  |  |
| t88 | 1 | 32 | *None* | **6** | **31** | **63** |  |  |  |  |  |  |
| p19 | 1 | 67 | I-F (100) |  |  | **75** | **19** | **6** |  |  |  |  |
| p71 | 1 | 62 | *None* |  | **31** | **44** | **6** | **13** | **6** |  |  |  |
| p37 | 1 | 34 | *None* |  | **13** | **81** | **6** |  |  |  |  |  |
| t75 | 1 | 28 | *None* |  | **19** | **81** |  |  |  |  |  |  |
| t73 | 1 | 37 | I-F (100) |  |  | **6** | **56** | **32** | **6** |  |  |  |
| t166 | 1 | 45 | *None* |  |  | **19** | **81** |  |  |  |  |  |
| t24 | 1 | 28 | I-F (100) |  |  | **62** | **38** |  |  |  |  |  |
| p18 | 1 | 33 | I-F (100), I-C (100) |  | **19** | **81** |  |  |  |  |  |  |
| t148 | 1 | 60 | I-F (100) |  | **25** | **75** |  |  |  |  |  |  |
| t30 | 1 | 50 | *None* |  | **38** | **62** |  |  |  |  |  |  |
| p85 | 1 | 74 | I-F (100) |  |  |  |  |  | **50** | **25** | **6** | **19** |
| t146 | 1 | 44 | *None* |  |  | **6** | **6** | **88** |  |  |  |  |
| t69 | 1 | 24 | I-F (100), I-E (100) |  |  |  | **6** | **81** | **13** |  |  |  |
| p68 | 1 | 34 | I-F (100) |  | **88** | **12** |  |  |  |  |  |  |
| t93 | 1 | 31 | I-F (100) |  | **62** | **38** |  |  |  |  |  |  |
| t91 | 1 | 28 | I-F (100) | **6** | **69** | **25** |  |  |  |  |  |  |
| p24 | 1 | 23 | I-F (7) |  |  | **71** | **29** |  |  |  |  |  |
| t52 | 1 | 61 | *None* |  |  | **6** | **47** | **47** |  |  |  |  |
| p64 | 1 | 10 | *None* | **6** | **25** | **69** |  |  |  |  |  |  |
| p62 | 1 | 70 | *None* |  |  | **69** | **31** |  |  |  |  |  |
| p45 | 1 | 75 | *None* |  |  |  | **6** | **38** | **56** |  |  |  |
| t32 | 1 | 58 | *None* |  |  |  |  | **88** | **12** |  |  |  |
| t106 | 1 | 57 | I-F (94) |  |  |  |  | **13** | **74** | **13** |  |  |
| t124 | 1 | 35 | I-F (100) |  | **69** | **31** |  |  |  |  |  |  |
| p25 | 1 | 66 | I-E (100) |  |  |  |  |  |  | **12** | **88** |  |
| p15 | 1 | 78 | *None* |  |  |  | **6** | **6** | **50** | **6** | **26** | **6** |
| t39 | 1 | 73 | *None* |  |  | **12** | **88** |  |  |  |  |  |
| p56 | 1 | 68 | I-E (100) |  |  |  |  | **6** | **81** | **13** |  |  |
| t162 | 1 | 31 | I-F (100) | **12** | **88** |  |  |  |  |  |  |  |
| t145 | 1 | 30 | *None* |  |  |  | **81** | **6** | **13** |  |  |  |
| p9 | 1 | 40 | *None* |  |  | **56** | **44** |  |  |  |  |  |
| t159 | 1 | 31 | I-F (100) |  | **6** | **56** | **38** |  |  |  |  |  |
| t95 | 1 | 79 | I-F (100) |  |  |  |  | **6** |  | **69** | **25** |  |
| t98 | 1 | 67 | I-F (100) |  |  |  | **38** | **62** |  |  |  |  |
| t167 | 1 | 66 | I-E (100) |  |  |  | **31** | **69** |  |  |  |  |
| p84 | 1 | 17 | *None* | **38** | **62** |  |  |  |  |  |  |  |
| t36 | 1 | 32 | I-F (100) |  | **62** | **38** |  |  |  |  |  |  |
| p16 | 1 | 31 | I-F (100), I-E (100) |  | **19** | **81** |  |  |  |  |  |  |
| p39 | 1 | 24 | I-F (100) |  | **12** | **88** |  |  |  |  |  |  |
| p6 | 1 | 26 | *None* |  |  |  | **56** | **44** |  |  |  |  |
| t160 | 1 | 23 | I-F (100) |  | **19** | **81** |  |  |  |  |  |  |
| p17 | 1 | 31 | I-E (100) |  | **6** | **88** | **6** |  |  |  |  |  |
| t112 | 1 | 65 | I-F (100) |  |  |  | **13** | **7** | **80** |  |  |  |
| t111 | 1 | 20 | I-F (100) |  | **69** | **31** |  |  |  |  |  |  |
| t117 | 1 | 36 | I-E (100) | **81** | **19** |  |  |  |  |  |  |  |
| t169 | 1 | 36 | I-C (100) | **88** | **12** |  |  |  |  |  |  |  |
| t48 | 1 | 6 | I-F (100) |  | **50** | **44** | **6** |  |  |  |  |  |
| p36 | 1 | 31 | I-F (100), I-C (100) |  | **31** | **69** |  |  |  |  |  |  |
| p48 | 1 | 69 | I-F (100) |  |  |  | **75** | **25** |  |  |  |  |
| t108 | 1 | 59 | *None* |  |  |  | **88** | **12** |  |  |  |  |
| t3 | 1 | 39 | *None* |  |  | **12** | **88** |  |  |  |  |  |
| t63 | 2 | 70 | *None* |  |  | **31** | **50** | **19** |  |  |  |  |
| t16 | 2 | 42 | *None* |  |  | **56** | **38** | **6** |  |  |  |  |
| t114 | 2 | 62 | I-E (100) |  | **56** | **44** |  |  |  |  |  |  |
| t83 | 2 | 51 | I-E (100) |  |  | **26** | **31** | **31** | **6** | **6** |  |  |
| t100 | 2 | 59 | *None* |  |  |  | **75** | **25** |  |  |  |  |
| p20 | 2 | 46 | I-F (100) |  |  |  | **13** | **31** |  | **56** |  |  |
| t74 | 2 | 41 | *None* |  |  |  | **12** | **88** |  |  |  |  |
| p55 | 2 | 61 | *None* |  |  |  | **12** | **44** | **44** |  |  |  |
| t62 | 2 | 69 | *None* |  |  |  | **12** | **88** |  |  |  |  |
| p22 | 2 | 44 | *None* |  |  | **12** | **88** |  |  |  |  |  |
| p58 | 3+ | 26 | *None* |  | **6** | **25** | **69** |  |  |  |  |  |
| t97 | 3+ | 37 | I-F (100) |  |  | **6** | **6** | **88** |  |  |  |  |
| p51 | 3+ | 35 | I-F (100) |  |  |  | **6** | **88** | **6** |  |  |  |

**
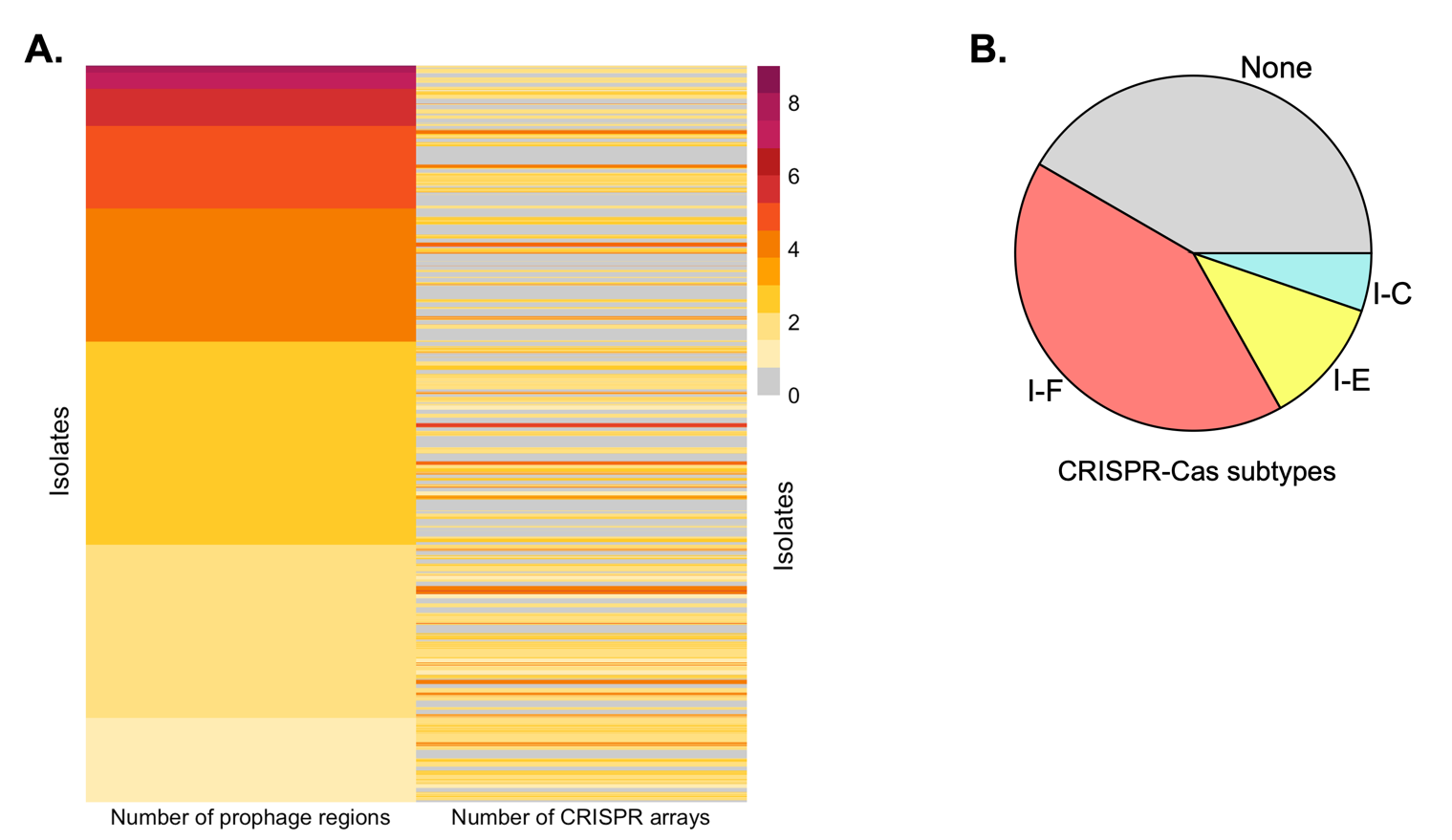
**

**Figure S3. (A)** The left column shows the number of prophage regions detected using VirSorter in each *Pseudomonas aeruginosa* isolate from people with bronchiectasis, and the right column shows the number of CRISPR arrays in functional CRISPR-Cas operons identified in each isolate using CRISPRCasTyper. The colour represents the total number (see key), and each isolate is represented by a row. **(B)** The number of patients that have isolates with each of the detected, functional CRISPR-Cas subtypes. Those labelled as ‘None’ represent patients where there were no intact, functional CRISPR-Cas subtypes detected in their sequenced isolates.

**
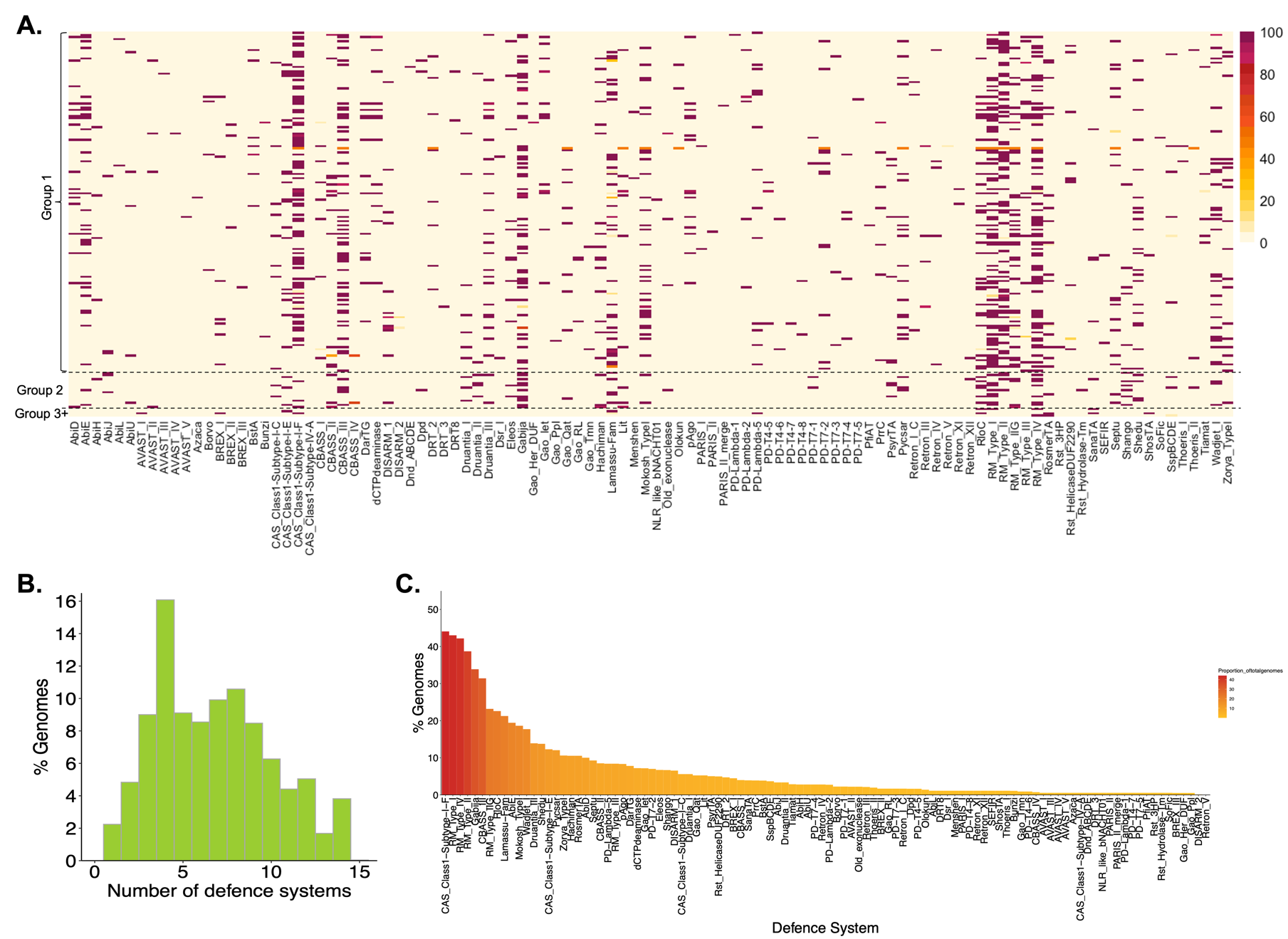
**

**Figure S4.** Defence systems in *Pseudomonas aeruginosa* isolates from patients with bronchiectasis, identified using DefenseFinder. This package screens for all possible defence systems and will also report non-functional CRISPR-Cas subtypes (additional to those reported in Figure S3). **(A)** The presence/absence of defence systems. Each row represents a patient, and the fill colour shows the percentage of isolates from each patient with the system (see key). Groups shown are the phylogenetic groups. **(B)** The frequency of defence systems in each isolates’ genome. **(C)** The percentage of isolate genomes found to have each defence system. The fill represents proportion of all isolates with the system (see key).

**Figure S5. (A)** The number of genes in the core (pink) and accessory (blue and yellow) *Pseudomonas aeruginosa* genome in the pangenome of ~16 isolates sequenced per bronchiectasis patient, which are predominantly all the same strain (98% of patients). **(B)** The number of genes in the *P. aeruginosa* accessory genome of the pangenome of individual patients’ sequenced population (~16 isolates), in the same order as A. In both graphs each bar represents a patient, ordered by phylogenetic group. The patients with plasmids detected are labelled with the replicon and the number in brackets indicates how many sequenced isolates carried the replicon/s from that patient.

**Table S8.** The genes that most frequently carried non-synonymous single nucleotide polymorphisms (SNPs) between *Pseudomonas aeruginosa* isolates from the same bronchiectasis patient amongst either phylogenetic group 1 (PAO1-like) or group 2 (PA14-like). The total number of patients shows number of patients across both groups that have polymorphism in the gene. The proportion of isolates column bar charts show the proportion of sequenced isolates per patient with a non-synonymous mutation in the gene; the blue shows isolates with a mutation (top bar) and the grey shows isolates without a mutation (bottom bar), the y axis is the number of isolates.

| **Gene name** | **Gene product** | **Total number of patients** | **Proportion of isolates** |
| --- | --- | --- | --- |
| *prc* | Periplasmic tail-specific protease | 21 |  |
| *opmH* | Probable outer membrane protein precursor | 20 |  |
| *algU* | Sigma factor | 20 |  |
| PA2217 | Probable aldehyde dehydrogenase | 16 |  |
| *flgK* | Flagellar hook-associated protein 1 | 16 |  |
| PA1874 | Hypothetical protein | 16 |  |
| *fptA* | Fe(III)-pyochelin outer membrane receptor precursor | 14 |  |
| *fliC* | Flagellin type B | 13 |  |
| *pilB* | Type 4 fimbrial biogenesis protein | 12 |  |
| *vgrG3* | VgrG3 | 12 |  |
| *mucA* | Anti-sigma factor | 11 |  |
| PA1572 | Hypothetical protein | 11 |  |
| *xcpQ* | General secretion pathway protein D | 6 |  |
| PA14_55400 | Hypothetical protein | 5 |  |
| *oprE* | Anaerobically-induced outer membrane porin OprE precursor | 5 |  |
| PA2590 | Hypothetical protein | 5 |  |
| *traG* | Conjugal transfer coupling protein | 3 |  |

**Figure S6.** *Pseudomonas aeruginosa* isolates from the one bronchiectasis patient without an OXA-50-like beta-lactamase (region shown by the red box) due to the large deletion shown.
